# Radiomics in predicting treatment response in non-small-cell lung cancer: current status, challenges and future perspectives

**DOI:** 10.1007/s00330-020-07141-9

**Published:** 2020-08-18

**Authors:** Madhurima R. Chetan, Fergus V. Gleeson

**Affiliations:** 1 Department of Radiology, Churchill Hospital, Oxford University Hospitals NHS Foundation Trust, Old Road, Headington, Oxford, OX3 7LE UK; 2 Nuffield Department of Surgical Sciences, John Radcliffe Hospital, University of Oxford, Room 6607, Level 6, Oxford, OX3 9DU UK; 3 Department of Oncology, Old Road Campus Research Building, University of Oxford, Roosevelt Drive, Oxford, OX3 7DQ UK

**Keywords:** Carcinoma, non-small-cell lung, Tomography, X-ray computed, Positron emission tomography computed tomography, Biomarkers, Precision medicine

## Abstract

**Objectives:**

Radiomics is the extraction of quantitative data from medical imaging, which has the potential to characterise tumour phenotype. The radiomics approach has the capacity to construct predictive models for treatment response, essential for the pursuit of personalised medicine. In this literature review, we summarise the current status and evaluate the scientific and reporting quality of radiomics research in the prediction of treatment response in non-small-cell lung cancer (NSCLC).

**Methods:**

A comprehensive literature search was conducted using the PubMed database. A total of 178 articles were screened for eligibility and 14 peer-reviewed articles were included. The radiomics quality score (RQS), a radiomics-specific quality metric emulating the TRIPOD guidelines, was used to assess scientific and reporting quality.

**Results:**

Included studies reported several predictive markers including first-, second- and high-order features, such as kurtosis, grey-level uniformity and wavelet HLL mean respectively, as well as PET-based metabolic parameters. Quality assessment demonstrated a low median score of + 2.5 (range − 5 to + 9), mainly reflecting a lack of reproducibility and clinical evaluation. There was extensive heterogeneity between studies due to differences in patient population, cancer stage, treatment modality, follow-up timescales and radiomics workflow methodology.

**Conclusions:**

Radiomics research has not yet been translated into clinical use. Efforts towards standardisation and collaboration are needed to identify reproducible radiomic predictors of response. Promising radiomic models must be externally validated and their impact evaluated within the clinical pathway before they can be implemented as a clinical decision-making tool to facilitate personalised treatment for patients with NSCLC.

**Key Points:**

*• The included studies reported several promising radiomic markers of treatment response in lung cancer; however, there was a lack of reproducibility between studies.*

*• Quality assessment using the radiomics quality score (RQS) demonstrated a low median total score of + 2.5 (range − 5 to + 9).*

*• Future radiomics research should focus on implementation of standardised radiomics features and software, together with external validation in a prospective setting.*

## Introduction

Radiomics is the extraction of data from medical imaging using mathematical algorithms for advanced image analysis [1]. The concept underlying radiomics is that medical imaging contains quantitative information, which is not discernible by the human eye, and which may reflect the underlying pathophysiology of the tissue. In cancer imaging, quantitative radiomic features have the potential to characterise tumour phenotype. An important aim of radiomics is to construct predictive models for treatment response, based on the tumour phenotype characteristics derived from medical images. This is essential for the pursuit of personalised medicine, in which treatment is tailored based on the characteristics of individual patients and their tumours.

Worldwide, lung cancer is the most common cancer and the leading cause of cancer death. In 2018, 2.09 million people were diagnosed with lung cancer and there were 1.76 million deaths from lung cancer [2]. Non-small-cell lung carcinoma (NSCLC) is the most frequent type of lung cancer, accounting for 87% of all lung cancer diagnoses [3]. Multiple treatment modalities are used in NSCLC: surgery; radiotherapy, including stereotactic ablative radiotherapy; and systemic therapy, including cytotoxic chemotherapy, tyrosine kinase inhibitors and immune checkpoint inhibitors [4]. Patients with NSCLC have baseline computed tomography (CT) and/or fluorodeoxyglucose positron emission tomography/computed tomography (FDG PET/CT) imaging for diagnosis and staging. Regular follow-up imaging is also performed to evaluate treatment response and monitor for recurrence.

Pathologic or radiologic criteria can be used to assess treatment response. Pathologic response is a ‘hard’ endpoint; however, it can only be evaluated in the 16% of patients with NSCLC who undergo surgical resection [5]. Radiologic response is therefore the mainstay of treatment response assessment in NSCLC. Response Evaluation Criteria in Solid Tumours (RECIST) provides an objective, standardised method for reporting response to therapy based on unidimensional evaluation of tumour size [6]. RECIST criteria are embedded in the definition of oncology trial endpoints, such as response rate and progression-free survival [7]. In clinical practice, the radiologic evaluation of treatment response largely relies on tumour size, supplemented with a qualitative assessment of other tumour characteristics such as homogeneity and shape.

From a quantitative viewpoint, this approach is not only basic but also ignorant of a substantial amount of information within the medical image. The radiomics approach has the potential to identify quantitative markers of treatment response earlier in the course of treatment. This can enable treatment to be adapted, intensified or altered earlier in the course of disease in order to improve patient outcomes.

Although radiomics shows great promise, it has not yet been translated into clinical practice [8]. In this literature review, we summarise the current applications of radiomics in the prediction of treatment response in NSCLC and evaluate the scientific and reporting quality of studies in this field. Other reviews in this field have focused on novel radiomic techniques [9] and predicting prognosis [10]; however, our focus is on the prediction of treatment response earlier in the course of treatment, and, to our knowledge, this is the first paper evaluating the quality of research in this field. We discuss the research challenges underlying the translational gap in radiomics and postulate on future directions.

## Materials and methods

### Search strategy and study selection

A comprehensive literature search was conducted using the PubMed database using a wide range of keywords and Medical Subject Headings (MeSH) terms. Full details of the search strategy are provided in Table 1.

**Table 1:** Keywords and MeSH terms used to search the PubMed database

Search	Query	Records found
#2	Search ((“Carcinoma, Non-Small-Cell Lung”[Mesh]) AND ((((computed tomography) OR ct)) AND (((quantitative) OR texture) OR feature))) AND ((((response) OR outcome) OR prognosis) OR survival) Sort by: [pubsolr12]	364
#8	Search ((“Carcinoma, Non-Small-Cell Lung”[Mesh]) AND ((((computed tomography) OR ct)) AND (((quantitative) OR texture) OR feature))) AND ((((response) OR outcome) OR prognosis) OR survival)	201
#23	Search ((“Carcinoma, Non-Small-Cell Lung”[Mesh]) AND ((((computed tomography) OR ct)) AND (((quantitative) OR texture) OR feature))) AND (response)	79
#24	Search “Carcinoma, Non-Small-Cell Lung”[Mesh]	48880
#27	Search “Tomography, X-Ray Computed”[Mesh]	409564
#30	Search “Positron-Emission Tomography”[Mesh]	55740
#33	Search “Positron Emission Tomography Computed Tomography”[Mesh]	6695
#34	Search ((“Tomography, X-Ray Computed”[Mesh]) OR “Positron-Emission Tomography”[Mesh]) OR “Positron Emission Tomography Computed Tomography”[Mesh]	441279
#35	Search (((quantitative) OR texture) OR feature) OR radiomic*	982225
#37	Search ((((((((disease response) OR treatment response) OR therapy response) OR tumor volume) OR tumour volume) OR tumor size) OR tumour size) OR tumor shrinkage) OR tumour shrinkage	1422709
#38	Search ((disease) OR treatment) OR therapy	12737365
#39	Search ((((disease) OR treatment) OR therapy)) AND response	1217708
#40	Search (tumor) OR tumour	3786725
#41	Search (((size) OR volume) OR shrinkage) OR response	3770284
#42	Search (((tumor) OR tumour)) AND ((((size) OR volume) OR shrinkage) OR response)	634912
#43	Search ((((((disease) OR treatment) OR therapy)) AND response)) OR ((((tumor) OR tumour)) AND ((((size) OR volume) OR shrinkage) OR response))	1534582
#44	Search (((“Carcinoma, Non-Small-Cell Lung”[Mesh]) AND (((“Tomography, X-Ray Computed”[Mesh]) OR “Positron-Emission Tomography”[Mesh]) OR “Positron Emission Tomography Computed Tomography”[Mesh])) AND ((((quantitative) OR texture) OR feature) OR radiomic*)) AND (((((((disease) OR treatment) OR therapy)) AND response)) OR ((((tumor) OR tumour)) AND ((((size) OR volume) OR shrinkage) OR response)))	178

The search was performed in August 2019. Search #44 returned 178 results and was used in this review

*MeSH*, medical subject headings

**Fig. 1:**
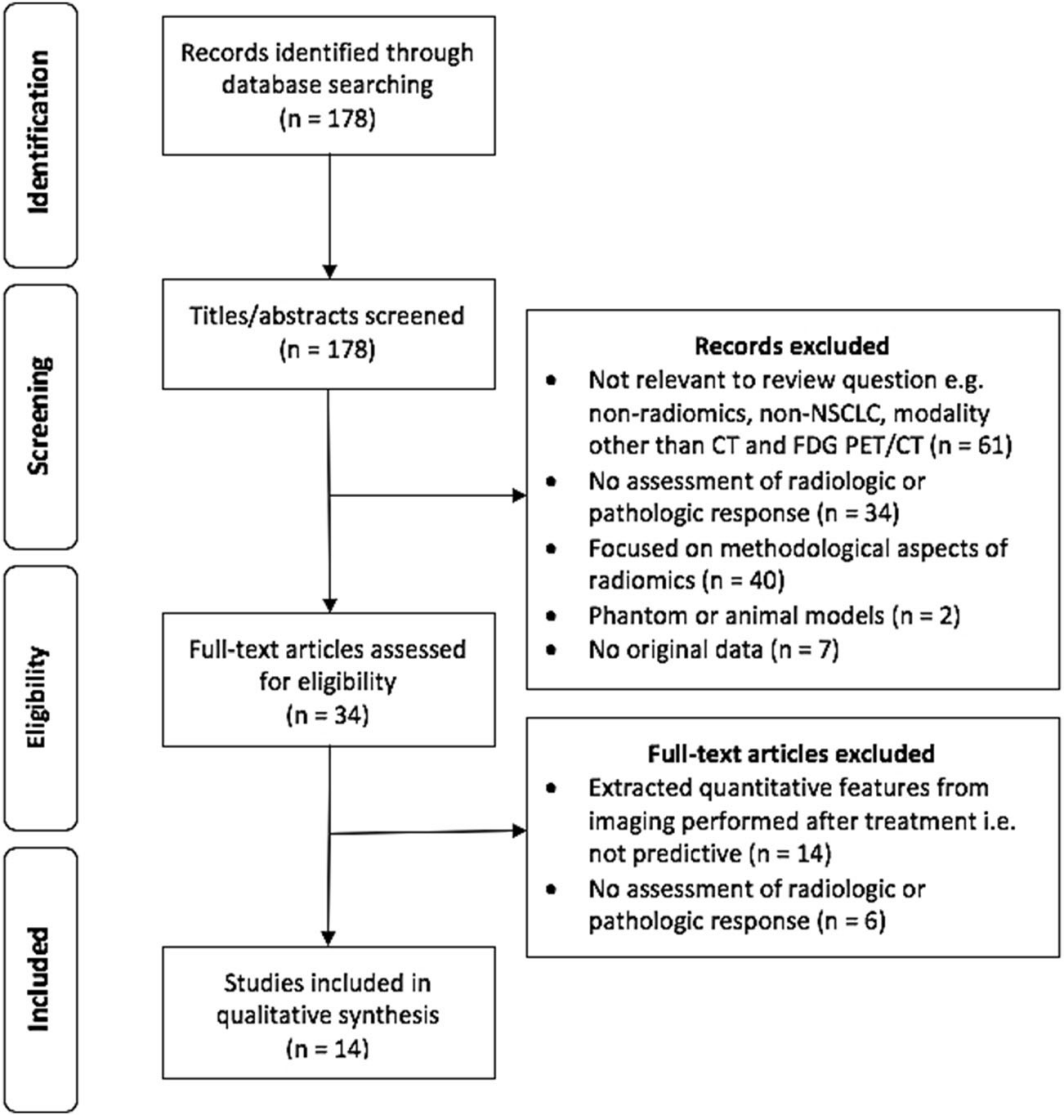
Flow diagram of the study selection process

We included all studies which evaluated quantitative features extracted from baseline or early treatment CT or PET/CT scans against treatment response in patients undergoing treatment of any modality for NSCLC of any stage. We applied the following exclusion criteria: (1) studies not assessing radiologic or pathologic response as an endpoint; (2) studies focussed purely on methodological aspects of radiomics; (3) studies extracting quantitative features from imaging performed after treatment, i.e. not predictive; (4) studies in phantom or animal models; (5) articles without original data, such as reviews and editorials. No study was excluded based on language, geographical location or date of publication. A total of 178 titles/abstracts were screened, and 34 eligible studies were retrieved as full text. Fourteen peer-reviewed articles published from 2003 to 2018 were included in this review. Full details of screening and eligibility assessment are provided in Fig. 1.

### Data extraction and analysis

The following data were extracted from each included study: sample size, NSCLC stage, treatment, follow-up duration, imaging modality, quantitative feature(s), treatment response endpoint and performance metrics.

The studies were systematically evaluated using the radiomics quality score (RQS) [11]. The RQS is a radiomics-specific quality assessment tool which emulates the transparent reporting of a multivariable prediction model for individual prognosis or diagnosis (TRIPOD) reporting guidelines [12]. The RQS comprises 16 components and is defined in full elsewhere [11].

In this review, each study was assigned a number of points per RQS component and summed to give an overall score (range − 8 to + 36). Studies were scored on their methodology pertaining to the treatment response endpoint, and not any other endpoints explored within the study. Certain components of RQS were interpreted as follows. Where mention was made of a standardised imaging protocol but image acquisition parameters were not provided in the main text, supplementary information or referenced paper, imaging was not reproducible and therefore, a score of 0 was assigned for ‘image protocol quality’. Where a single or very small number of quantitative features were tested, the risk of overfitting was minimal and therefore, a score of + 3 was given for ‘feature reduction or adjustment for multiple testing’. Where cross-validation or nested cross-validation was performed using only the training data, true validation without re-training was missing and therefore, a score of − 5 was assigned for ‘validation’. Where potential utility was discussed without any analysis of impact on health outcomes, clinical utility was not demonstrated and therefore, a score of 0 was awarded for ‘potential clinical utility’.

## Results

A summary of the imaging predictors of treatment response in NSCLC is provided in Table 2.

**Table 2:** Imaging predictors of treatment response

Study	NSCLC stage	Treatment modality	Sample size	Imaging modality	Number of features	Result	RQS
Predicting pathologic response							
Coroller et al, 2017 [13]	IIA-IIIB	Chemoradiotherapy + surgical resection	85	CT	20	A model built from ten primary tumour and ten lymph node baseline radiomic features (AUC 0.68, *p* value < 0.05)	7
Coroller et al, 2016 [14]	IIA-IIIB	Chemoradiotherapy + surgical resection	127	CT	15	Baseline wavelet HLL mean (AUC = 0.63, *p* value = 0.01)	7
Chong et al, 2014 [15]	IIIA	Erlotinib + surgical resection; Concurrent chemoradiotherapy + surgical resection	23; 28	CT; CT	7	Baseline intensity variability (adjusted OR 1.093, 95% CI 1.009–1.184, *p* = 0.028); Baseline kurtosis (adjusted OR 1.107, 95% CI 1.026–1.195, *p* = 0.009)	1
Aukema et al, 2010 [16]	I-III	Erlotinib + surgical resection	23	PET/CT	1	PET EORTC criteria for change in SUV_max_ (*κ*-agreement 0.55, *p* = 0.008)	8
Predicting RECIST response							
Dong et al, 2016 [17]	III	Concurrent chemoradiotherapy	58	PET/CT	30	Change in contrast (AUC 0.86), and coefficient of variation of SUV (AUC 0.80)	3
Cook et al, 2015 [18]	IIIB-IV	Erlotinib	47	PET/CT	42	Change in SUV_max_ (*p* = 0.007), standard deviation (*p* = 0.01), first-order entropy (*p* = 0.005) and uniformity (*p* = 0.008)	1
Keam et al, 2015 [19]	IIIB-IV, EGFR-mutated	Gefitinib	75	PET/CT	2	Baseline SUV_max_ (*p* = 0.793), and TLG (*p* = 0.274)	-1
Cook et al, 2013 [20]	IB-IIIB	Concurrent chemoradiotherapy	53	PET/CT	9	Baseline coarseness (AUC 0.80, *p* = 0.003), contrast (AUC 0.82, *p* = 0.002) and busyness (AUC 0.72, *p* = 0.027)	-4
Ravanelli et al, 2013 [21]	IIIB-IV	Chemotherapy	53	CT	3	Baseline grey-level uniformity in adenocarcinoma subgroup (AUC 0.741, *p* < 0.01)	3
Ohno et al, 2012 [22]	III	Concurrent chemoradiotherapy	64	PET/CT	1	Baseline SUV_max_ (AUC 0.64)	9
Weber et al, 2003 [23]	IIIB-IV	Chemotherapy	57	PET/CT	9	Change in SUV (AUC 0.91) and FDG net-influx constant (AUC 0.92)	3
Predicting tumour volume							
Ramella et al, 2018 [24]	III	Concurrent chemoradiotherapy	91	CT	12	A model built from five clinical features (sex, nodal status, histology, EGFR mutation, smoking) and seven baseline radiomic features (two grey-level co-occurrence matrix measures, four local binary patterns-TOP measures, one statistical measure) (AUC 0.82, 95% CI 0.73–0.91)	1
Zhang et al, 2018 [25]	≥ II	Radiotherapy	34	CT	Unknown	A model of tumour shrinkage built with mid-course correction (AUC 0.85)	2
Hunter et al, 2015 [26]	II-III	Radiotherapy	64	CT	45	A model built from 35 baseline radiomic features (*p* = 4.36 × 10^−9^)	-5

The RQS ranges from − 8 to 36; a higher RQS implies a thoroughly described and researched radiomics model

*AUC*, area under the receiver operating characteristic curve; *CI*, confidence interval; *CT*, computed tomography; *EGFR*, epidermal growth factor receptor; *EORTC*, European Organization for Research and Treatment of Cancer; *FDG*, fluorodeoxyglucose; *NSCLC*, non-small-cell lung cancer; *OR*, odds ratio; *PET*, positron emission tomography; *RQS*; radiomics quality score; *SUV*, standardised uptake value; *SUV*_*max*_, maximum standardised uptake value; *TOP*, three orthogonal planes

### Predicting pathologic response

Pathologic complete response is defined as the absence of tumour cells in all specimens. It is an important prognostic factor in locally advanced NSCLC and is associated with greater overall survival and lower rates of local and distant recurrence [27].

Three retrospective studies investigated whether CT-based radiomic features predict pathologic response.

In patients with NSCLC treated with combination chemoradiotherapy followed by surgical resection, wavelet HLL mean, a high-order texture feature, was a moderate predictor of pathologic complete response (AUC 0.63, *p* = 0.01) [14].

A further study by the same group showed that lymph node texture features better predicted pathologic complete response than primary tumour texture features [13]. A model built from ten primary tumour and ten lymph node radiomic features was significantly better at predicting pathologic response than conventional features (AUC 0.68, *p* < 0.05), whilst a combined clinical and radiomic model was best at predicting gross residual disease (AUC 0.73, *p* < 0.05) [13].

Chong et al (2014) performed a multivariate analysis in two patient cohorts receiving combination chemoradiotherapy and tyrosine kinase inhibitor therapy respectively, followed by surgical resection. Pathologic response was independently predicted by kurtosis in patients receiving combination chemoradiotherapy (OR 1.107, *p* = 0.009), and by intensity variability in patients receiving tyrosine kinase inhibitor therapy (OR 1.093, *p* = 0.028) [15].

Aukema et al (2010) performed a prospective study investigating the relationship between PET-based quantitative features and pathologic response in patients with NSCLC receiving combination chemoradiotherapy followed by surgical resection. Early change in maximum standardised uptake value (SUV_max_) was an excellent predictor of pathologic complete response (*κ*-agreement 0.55, *p =* 0.008) [16].

### Predicting radiologic response

Radiologic evaluation with RECIST criteria is widely used as a marker of treatment response in oncology trials. A recent study of 23,259 patients with cancer (36% lung cancer) treated with chemotherapy and/or targeted therapies demonstrated a linear relationship between change in unidimensional tumour size and overall survival [28]. Change in tumour volume is another effective tool for evaluating radiologic response after treatment, which has been shown to better correlate with pathologic complete response than unidimensional RECIST in patients with locally advanced NSCLC [29].

PET-based metabolic parameters were investigated by four studies. In locally advanced or metastatic NSCLC treated with tyrosine kinase inhibitors, baseline PET metabolic parameters, such as SUV_max_ and total lesion glycolysis (TLG), did not correlate with RECIST response [18, 19]. However, a change in SUV_max_ over serial PET imaging from baseline to 6 weeks into treatment was predictive of RECIST response at 12 weeks (*p* = 0.007) [18]. Similar results were demonstrated in patients receiving concurrent chemoradiotherapy. In this group, baseline SUV_max_ was not a good predictor of RECIST response (AUC 0.64) [22]. A prospective study demonstrated that change in SUV_max_ and FDG net-influx constant (Ki) over serial PET imaging from baseline to first cycle of chemotherapy was an excellent predictor of RECIST response (AUC 0.91 and 0.92 respectively) [23].

Three studies investigated PET-based texture and heterogeneity parameters. In locally advanced or metastatic NSCLC treated with tyrosine kinase inhibitor therapy, features reflecting tumour heterogeneity at baseline PET/CT imaging, such as first-order standard deviation, entropy and uniformity, were associated with RECIST response (*p <* 0.01) [18]. In patients on concurrent chemoradiotherapy, baseline texture features such as contrast, coarseness and busyness were predictive of RECIST response (AUC 0.80, 0.82 and 0.72 respectively, *p* < 0.03) [20]. Another study showed that contrast and coefficient of variation of SUV at baseline (AUC 0.80 and 0.78 respectively) as well as change in contrast and coefficient of variation of SUV over serial PET imaging from baseline to 4 weeks into treatment (AUC 0.86 and 0.80 respectively) both predicted RECIST response at 12 weeks [17].

In locally advanced or metastatic NSCLC treated with first-line platinum-based chemotherapy, grey-level uniformity at baseline CT was predictive of RECIST response in a subgroup where the histology was adenocarcinoma (AUC 0.741, *p* < 0.01) [21].

Ramella et al (2018) showed that seven radiomic features extracted from pre-treatment CT images, in combination with five conventional clinical features, predicted tumour volume after completion of concurrent chemoradiotherapy (AUC 0.82) [24].

Other groups have built predictive models with the aim of facilitating adaptive radiotherapy. A model built from 35 quantitative features extracted from pre-treatment CT images was a good predictor of tumour volume after 6 weeks of radiotherapy (R 0.83) [26]. Zhang et al (2018) showed that a model using pre-treatment CT features in combination with mid-treatment CT for ‘mid-course correction’, and validated in an independent cohort, was also a good predictor of post-radiotherapy tumour volume (AUC 0.85) [25].

### Scientific and reporting quality

The median total score for the fourteen included studies was + 2.5 (range − 5 to + 9) on a scale where the maximum available score is + 36. Adherence to the RQS evaluation criteria and reporting guidelines is summarised in Table 3.

**Table 3:** Quality assessment using the RQS

RQS component (score range)	Image protocol quality (0 to 2)	Multiple segmentations (0 to 1)	Phantom study on all scanners (0 to 1)	Imaging at multiple time points (0 to 1)	Feature reduction or adjustment for multiple testing (-3 to 3)	Multivariable analysis with non-radiomic features (0 to 1)	Detect and discuss biological correlates (0 to 1)	Cut-off analyses (0 to 1)	Discrimination statistics (0 to 2)	Calibration statistics (0 to 2)	Prospective study registered in a trial database (0 to 7)	Validation (-5 to 5)	Comparison to gold standard (0 to 2)	Potential clinical utility (0 to 2)	Cost-effectiveness analysis (0 to 1)	Open science and data (0 to 4)
Coroller et al, 2017 [27]	*0*	**1**	*0*	**1**	**3**	**1**	**1**	*0*	**2**	*0*	*0*	*− 5*	**2**	*0*	*0*	***1***
Coroller et al, 2016 [12]	*0*	**1**	*0*	**1**	**3**	**1**	**1**	*0*	**2**	*0*	*0*	*− 5*	**2**	*0*	*0*	***1***
Chong et al, 2014 [10]	***1***	*0*	*0*	*0*	**3**	*0*	**1**	*0*	***1***	*0*	*0*	*− 5*	*0*	*0*	*0*	*0*
Aukema et al, 2010 [16]	***1***	*0*	*0*	*0*	**3**	*0*	**1**	***1***	*0*	*0*	**7**	*− 5*	*0*	*0*	*0*	0
Dong et al, 2016 [26]	***1***	**1**	*0*	*0*	**3**	**1**	**1**	*0*	***1***	*0*	*0*	*− 5*	*0*	*0*	*0*	*0*
Cook et al, 2015 [23]	***1***	**1**	*0*	*0*	**3**	*0*	**1**	*0*	*0*	*0*	*0*	*− 5*	*0*	*0*	*0*	*0*
Keam et al, 2015 [22]	*0*	*0*	*0*	*0*	**3**	*0*	**1**	*0*	*0*	*0*	*0*	*− 5*	*0*	*0*	*0*	*0*
Cook et al, 2013 [24]	***1***	*0*	*0*	*1*	*− 3*	*0*	**1**	*0*	***1***	*0*	*0*	*− 5*	*0*	*0*	*0*	*0*
Ravanelli et al, 2013 [25]	***1***	**1**	*0*	*0*	**3**	**1**	**1**	*0*	***1***	*0*	*0*	*− 5*	*0*	*0*	*0*	*0*
Ohno et al, 2012 [17]	***1***	**1**	*0*	*0*	**3**	*0*	**1**	*0*	***1***	*0*	**7**	*− 5*	*0*	*0*	*0*	*0*
Weber et al, 2003 [21]	***1***	*0*	*0*	*0*	*− 3*	*0*	**1**	***1***	***1***	*0*	**7**	*− 5*	*0*	*0*	*0*	*0*
Ramella et al, 2018 [30]	*0*	*0*	*0*	*0*	**3**	*0*	*0*	*0*	**2**	*0*	*0*	*− 5*	*0*	*0*	*0*	***1***
Zhang et al, 2018 [31]	***1***	*0*	*0*	*0*	*− 3*	*0*	*0*	*0*	***1***	*0*	*0*	***3***	*0*	*0*	*0*	*0*
Hunter et al, 015 [32]	***1***	*0*	*0*	*0*	*− 3*	*0*	*0*	*0*	*0*	**2**	*0*	*− 5*	*0*	*0*	*0*	*0*

A traffic light system has been used per individual RQS component: italicized where the lowest possible score has been assigned, bold where the highest possible score has been assigned and bold italics where some points have been assigned but not the highest possible score. A higher score implies a thoroughly described and researched radiomics model

*RQS*, radiomics quality score

There were three prospective studies (21%) and eleven retrospective studies.

Of the fourteen studies, ten (71%) reported well-documented image acquisition protocols, although none used publicly available protocols. Multiple segmentations by two readers or automatic segmentation were performed in six studies (42%). Three studies (21%) tested feature robustness to temporal variability using test-retest datasets. However, feature robustness to inter-scanner variation with phantom imaging was not performed by any study.

Ten studies (71%) adopted feature reduction or adjustment for multiple testing as appropriate, using a variety of statistical methods such as leave-one-out cross-validation and random forest classification. Four studies (29%) performed a multivariable analysis with non-radiomic features. Two studies (14%) performed a cutoff analysis using a pre-specified threshold. Ten studies (71%) reported discriminatory statistics, e.g. AUC; however, only three used a resampling method. A single study reported calibration statistics. Validation was missing in thirteen studies (93%) and only a single study performed validation with a single dataset from another institute. Three studies (21%) made their code or data publicly available; however, no study made their scans or region-of-interest segmentations open-source.

Eleven studies (79%) discussed biological correlates of the radiomic features described. Two studies (14%) compare the extent to which their proposed predictive factors are superior to the current ‘gold standard’ method of tumour staging. No study performed a clinical utility analysis or a cost-effectiveness analysis.

## Discussion

In this review, the results of fourteen studies investigating CT and PET/CT radiomic predictors of treatment response in NSCLC have been summarised. The scientific and reporting quality of these studies has been evaluated.

The included studies reported a variety of predictive markers, from histogram-based properties such as kurtosis [15] to second-order textural features such as grey-level uniformity [21] to high-order features such as wavelet HLL mean [14] to features that describe changes in the PET-based net-influx rate constant (Ki) [23].

The same radiomic feature was rarely identified as being predictive of treatment response in NSCLC by more than one study. This is partly explained by the extensive heterogeneity between individual studies. The studies were carried out in different patient populations with different cancer stages being managed at different institutions with different timescales for follow-up imaging. Image acquisition and reconstruction protocols vary across institutions, introducing changes in quantitative imaging features that are not due to underlying biological reasons. The studies investigated disease response to several treatment modalities, including conventional radiotherapy, cytotoxic chemotherapy, concurrent chemoradiotherapy and tyrosine kinase inhibitors. Given their different mechanisms of action, it is biologically plausible that the radiomic markers of response are different for each modality. Chong et al (2014) studied patients with stage IIIA NSCLC at a single institution and identified different radiomic predictors of pathological response in those treated with chemotherapy and those treated with tyrosine kinase inhibitors [15]. To our knowledge, the radiomic predictors of pathologic or radiologic response in NSCLC treated with immunotherapy has not be studied.

Study quality was variable, with the fourteen included studies scoring between − 5 and + 9 on the RQS metric, where the maximum possible score was + 36. The relatively low scientific and reporting quality of the included studies may have increased the likelihood of a false-positive association between radiomic features and treatment response being reported.

Only three studies were prospective, with the benefit of standardised cancer stage, treatment and follow-up [16, 22, 23]. Whilst many studies clearly reported their imaging protocols, few studies employed test-retest imaging, phantom imaging and multiple segmentation to assess feature robustness. Only features with high repeatability and high reproducibility can reliably reflect underlying tissue biology and therefore be used as predictors of treatment response. Two included studies reported that coarseness and contrast are good predictors of RECIST response with AUCs ≥ 0.80 [17, 20]; however, these two higher order features have been shown to be among the least reproducible radiomic features [30]. Due to these factors, the promising results reported by individual studies are at high risk of bias and may not be externally valid.

Appropriate feature reduction methods were used in some studies; e.g. Coroller et al (2016 and 2017) [13, 14] excluded highly correlated and non-reproducible features prior to analysis. Without appropriate feature reduction, some studies were highly susceptible to overfitting; e.g. Hunter et al (2015) built a model using 35 radiomic features with a sample size of 64 patients [26]. It is generally accepted that a minimum of ten patients per radiomic feature is required for a model to be generalisable [1]. Studies mostly reported the performance of radiomic markers using discrimination statistics, and under-utilised calibration statistics. Only two of the studies reporting cutoff analyses used a pre-specified threshold [16, 23]. Post hoc optimal cutoff selection in combination with a large number of candidate radiomic features has been shown to significantly increase the risk of type I error (76% probability) [32]. External validation was missing in all but one study [25]. Taken together, these factors have likely produced over-optimistic estimates of predictive performance in many included studies.

Efforts were made to correlate radiomic features to biological features; however, evidence for clinical applicability was critically lacking. No study carried out a clinical utility analysis or cost-effectiveness analysis. An evaluation of the added value of radiomics in comparison to the current ‘gold standard’ was missing in all but two studies [13, 14]. It is therefore unsurprising that the proposed radiomic predictors have not been translated into clinical practice.

The key strengths of this review are its broad literature coverage, clear summary of study results and use of a standardised quality assessment tool. An important limitation is that this is not a systematic review. It is possible that some relevant studies have not been included as other medical databases and the grey literature have not been searched. A meta-analysis could not be performed due to the heterogeneity between individual studies.

It has been argued that the traditional radiomics approach is inferior to artificial intelligence (AI) approaches such as deep learning with convolutional neural networks [31], as AI precludes the need for manual feature extraction and selection which may introduce a human bias. However, AI approaches require significantly larger datasets of annotated imaging records, and the logic behind the decisions remains a ‘black box’ [33]. Combining traditional radiomics with AI holds the promise of benefitting from the advantages of both techniques [34]. Radiologists have a crucial role to play in curating high-quality imaging datasets by using uniform and structured reporting lexicon in order to facilitate large cohort studies. In reality, this is challenging in day-to-day clinical practice.

## Conclusion

In the future, it will be important to establish reproducible and interpretable radiomic markers of treatment response in lung cancer. The image biomarker standardisation initiative (IBSI) has attempted to address the lack of standardisation in radiomics by defining standardised imaging biomarker nomenclature and producing tools for verifying radiomics software implementations [35]. For radiomic models to become a clinically meaningful tools, future studies need to adopt a standardised approach in compliance with the IBSI standard.

A move from hypothesis-generation towards hypothesis-testing is needed in future radiomics studies. The promising predictive features described in this review must be validated using datasets from different institutions to establish diagnostic accuracy across many populations. This requires a collaborative approach across institutions. Predictive models must be investigated prospectively within the clinical pathway to establish clinical benefit. By demonstrating reproducibility and clinical utility, radiomic models can prove their potential as a clinical decision-making tool that facilitates personalised treatment for patients with NSCLC.

## Abbreviations

95% CI: 95% Confidence interval
AI: Artificial intelligence
AUC: Area under the receiver operating characteristic curve
CT: Computed tomography
EGFR: Epidermal growth factor receptor
EORTC: European Organization for Research and Treatment of Cancer
FDG: Fluorodeoxyglucose
IBSI: Image biomarker standardisation initiative
MeSH: Medical Subject Headings
NSCLC: Non-small-cell lung cancer
OR: Odds ratio
PET/CT: Positron emission tomography/computed tomography
RECIST: Response evaluation criteria in solid tumours
ROI: Region-of-interest
RQS: Radiomics quality score
SUV: Standardised uptake value
SUV_max_: Maximum standardised uptake value
TLG: Total lesion glycolysis
TRIPOD: Transparent reporting of a multivariable prediction model for individual prognosis or diagnosis
